# Infliximab for Idiopathic Deep Cutaneous Vasculitis Refractory to Cyclophosphamide

**DOI:** 10.1155/2010/951850

**Published:** 2010-06-27

**Authors:** Marcelo Derbli Schafranski, Giuliano Doretto Campanari

**Affiliations:** ^1^Universidade Estadual de Ponta Grossa (UEPG), Rua Carlos Osternack, 111, 84040120, Ponta Grossa, Paraná, Brazil; ^2^Departament of Dermatology, Inovare Health Services, 84040120, Ponta Grossa, Paraná, Brazil

## Abstract

Cutaneous vasculitis can be classified as primary or idiopathic; or secondary, when it presents as a manifestation of connective tissue diseases, infections, drug reactions or malignancies. Although most of the idiopathic cases are self-limited and responsive to supportive measures and nonsteroidal anti-inflammatory drugs, potent immunosuppressants are sometimes required for the management of the refractory situations. Here we describe a case of a 32-year-old Caucasian female patient with history of idiopathic cutaneous deep vasculitis unresponsive to methotrexate, dapsone, and cyclophosphamide who was effectively treated with infliximab.

## 1. Introduction

Cutaneous vasculitis, a disease with an annual incidence rate ranging from 39.6 to 59.8 per million [1], can be classified as primary or idiopathic; or secondary, when it presents as a manifestation of connective tissue diseases, infections, drug reactions or malignancies [2]. Although most of the idiopathic cases are self-limited and responsive to supportive measures (limb elevation, warming, avoid standing) and nonsteroidal anti-inflammatory drugs, potent immunosuppressants are sometimes required for the management of the refractory situations [3].

We herein describe a case of a 32-year-old Caucasian female patient with history of idiopathic cutaneous deep vasculitis unresponsive to methotrexate, dapsone, and cyclophosphamide, who was effectively treated with infliximab.

## 2. Case Report

**Figure 1:**
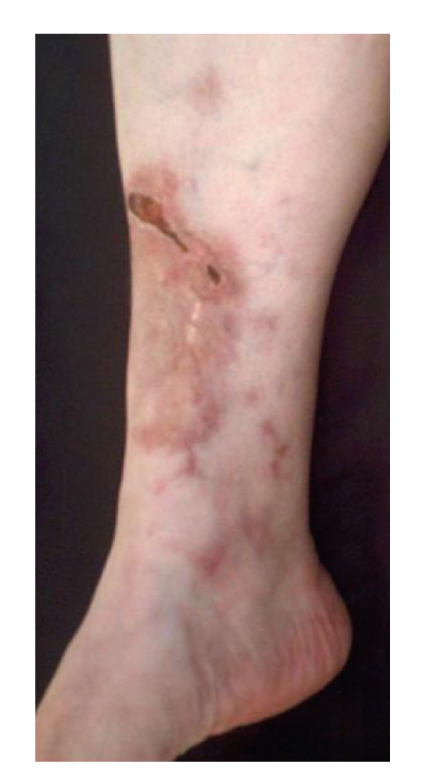
Vasculitic leg ulcer, in spite of the usage of high-dose prednisone (1 mg/kg/day).

**Figure 2:**
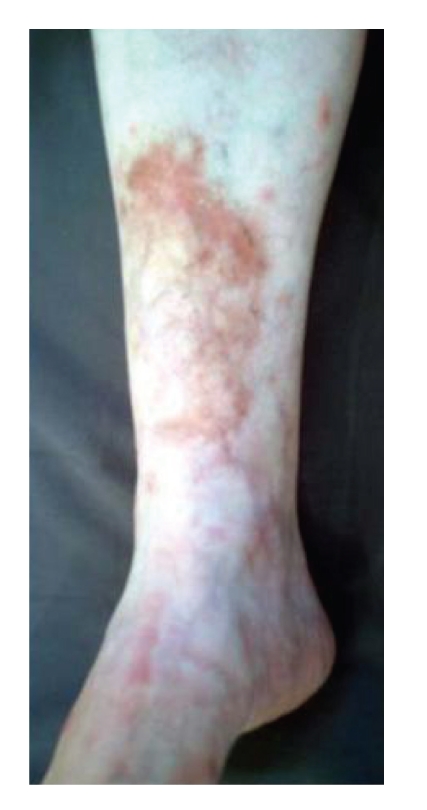
By the fourth infliximab infusion, the ulcers healed completely and allowed steroids withdrawal.

The patient felt well until September 2005, when palpable painful nodules and multiple ulcerated lesions developed in both legs and ankles. There was no history of arthritis, rashes, photosensitivity, other skin lesions, mouth ulcers, gastrointestinal, or urogenital symptoms. Antinuclear antibody (ANA), rheumatoid factor (RF), anti-neutrophil cytoplasmatic antibodies (ANCA), anti-RO, anti-LA, anti-HIV, HBsAg, HCV, antistreptolysin O (ASO), and cryoglobulins tested negative; complete blood count and a serum thyroid-stimulating hormone (TSH) determination were normal, as were a chest X-ray. Erythrocyte sedimentation rate (ESR) and C-reactive protein (CRP) were markedly elevated (90 mmHg on the first hour and 45 mg/dL—normal: 0–6 mg/dL; resp.), and there was a polyclonal gammopathy on a serum protein electrophoresis test. Examination of a skin smear sample to identify *Mycobacterium leprae *was negative. A cutaneous biopsy revealed fibrinoid degeneration of the perivascular and vessels walls collagen, as well as mononuclear and neutrophilic infiltrates extending to the deeper dermis and the presence of leukocytoclasia, corroborating the diagnosis of cutaneous deep vasculitis. The patient was given 60 mg/day of prednisone (approximately 1 mg/kg), with merely partial resolution of the signs and symptoms, even after 2 months of appropriate usage (Figure 1). As a steroid-sparing drug, methotrexate was given in a dosage up to 25 mg/week, unsuccessfully. Five monthly pulses of cyclophosphamide (0.75 g/m^2^/body surface) and dapsone 200 mg/day were also ineffective in reducing the oral prednisone dosage, and when the reduction was attempted, the vasculitis signs and symptoms recurred promptly. In August 2007, taking into consideration these therapeutic failures, the patient was started on infusions of 3 mg/kg infliximab on days 0, 15, and 45 and then every 8 weeks. As early as by the fourth infusion, prednisone dosage had already been tapered to zero and no vasculitis recurrence was observed (Figure 2). As of January 2009, no recurrences have occurred and no adverse drug effects were registered.

## 3. Discussion

About 40% of all cases of cutaneous vasculitis are idiopathic and, currently, no guidelines for its treatment are available. Most of the episodes are self-limiting, lasting only a few days or weeks, but in about 20% of cases, a chronic, unremitting course, takes place, when immunosuppressants (azathioprine, methotrexate, cyclophosphamide, cyclosporine, or mycophenolate mofetil) must be required [2].

Infliximab, a chimeric anti-TNF-*α* antibody, has been shown to be effective in a variety of inflammatory and autoimmune diseases, as rheumatoid arthritis, ankylosing spondylitis, and Crohn's disease [4]. Higher serum levels of the proinflammatory cytokines TNF-*α*, IL-1*β* has already been observed in small vessels cutaneous vasculitis patients when compared to healthy subjects [5], suggesting a possible role for these molecules in the pathogenesis of the disease. Reports published by Mang et al. [6] and Uthman et al. [7] also support the benefit of infliximab in cases of difficult-to-treat deep cutaneous vasculitis, even though in none of the cases reported by those authors cyclophosphamide was tried prior to the usage of biological therapy. Interestingly, McCain et al. [8] described a case of cutaneous vasculitis related to previous infliximab infusions, probably due to antidrug antibodies formation. Although infliximab is a drug that has been related to serious side effects, these can be properly monitored, identified, and managed, and its rapid onset of action certainly favors its use in refractory idiopathic deep cutaneous vasculitis.

## References

[1] Carlson JA, Ng BT, Chen K-R (2005). Cutaneous vasculitis update: diagnostic criteria, classification, epidemiology, etiology, pathogenesis, evaluation and prognosis. *American Journal of Dermatopathology*.

[2] Carlson JA, Cavaliere LF, Grant-Kels JM (2006). Cutaneous vasculitis: diagnosis and management. *Clinics in Dermatology*.

[3] Russell JP, Gibson LE (2006). Primary cutaneous small vessel vasculitis: approach to diagnosis and treatment. *International Journal of Dermatology*.

[4] Furst DE, Breedveld FC, Kalden JR (2007). Updated consensus statement on biological agents for the treatment of rheumatic diseases. *Annals of the Rheumatic Diseases*.

[5] Papi M, Didona B, De Pità O (1998). Livedo vasculopathy vs small vessel cutaneous vasculitis: cytokine and platelet P-selectin studies. *Archives of Dermatology*.

[6] Mang R, Ruzicka T, Stege H (2004). Therapy for severe necrotizing vasculitis with infliximab. *Journal of the American Academy of Dermatology*.

[7] Uthman IW, Touma Z, Sayyad J, Salman S (2005). Response of deep cutaneous vasculitis to infliximab. *Journal of the American Academy of Dermatology*.

[8] McCain ME, Quinet RJ, Davis WE (2002). Etanercept and infliximab associated with cutaneous vasculitis. *Rheumatology*.

